# Supplementary material for: Mechanistic insight into the role of AUXIN RESISTANCE4 in trafficking of AUXIN1 and LIKE AUX1-2
Source: Plant Physiol. 2023 Sep 30;194(1):422–33. doi: 10.1093/plphys/kiad506 (PMC10756756; doi:10.1093/plphys/kiad506)
Supplement: kiad506_Supplementary_Data [file kiad506_supplementary_data.zip › Suppemental Dataset S1/5445e4f6eaa2385e-AtAXR4/ss_report.pdf]

Phyre²

Email	charlie.hodgman@nottinghamac.uk
Description	AtAXR4___
Date	Tue Jan 10 14:09:39 GMT 2023
Unique Job ID	5445e4f6eaa2385e

Secondary structure and disorder prediction

1 . . . . 10 . . . . 20 . . . . 30 . . . . 40 . . . . 50 60

Sequence: M A I I T E E E E D P K T L N P P K N K P K D S D F T K S E S T M K N P K P Q S Q N P F P F W F Y F T V V V S L A T I I

Secondary structure

SS confidence

Disorder: ?

Disorder confidence

. . . . 70 . . . . 80 . . . . 90 . . . . 100 . . . . 110 120

Sequence: F I S L S L F S S Q N D P R S W F L S L P P A L R Q H Y S N G R T I K V Q V N S N E S P I E V F V A E S G S I H T E T V

Secondary structure

SS confidence

Disorder: ? ? ? ? ? ? ? ? ? ? ? ? ? ? ?

Disorder confidence

. . . . 130 . . . . 140 . . . . 150 . . . . 160 . . . . 170 180

Sequence: V I V H G L G L S S F A F K E M I Q S L G S K G I H S V A I D L P G N G F S D K S M V V I G G D R E I G F V A R V K E V

Secondary structure

SS confidence

Disorder: ? ? ? ? ? ?

Disorder confidence

. . . . 190 . . . . 200 . . . . 210 . . . . 220 . . . . 230 240

Sequence: Y G L I Q E K G V F W A F D Q M I E T G D L P Y E E I I K L Q N S K R R S F K A I E L G S E E T A R V L G Q V I D T L G

Secondary structure

SS confidence

Disorder: ? ? ? ? ? ? ? ?

Disorder confidence

. . . . 250 . . . . 260 . . . . 270 . . . . 280 . . . . 290 300

Sequence: L A P V H L V L H D S A L G L A S N W V S E N W Q S V R S V T L I D S S I S P A L P L W V L N V P G I R E I L L A F S F

Secondary structure

SS confidence

Disorder: ? ? ? ? ? ? ? ? ? ?

Disorder confidence

. . . . 310 . . . . 320 . . . . 330 . . . . 340 . . . . 350 360

Sequence: G F E K L V S F R C S K E M T L S D I D A H R I L L K G R N G R E A V V A S L N K L N H S F D I A Q W G N S D G I N G I

Secondary structure

SS confidence

Disorder: ? ? ? ? ? ? ? ? ? ? ? ? ? ? ? ?

Disorder confidence

. . . . 370 . . . . 380 . . . . 390 . . . . 400 . . . . 410 420

Sequence: P M Q V I W S S E A S K E W S D E G Q R V A K A L P K A K F V T H S G S R W P Q E S K S G E L A D Y I S E F V S L L P K

Secondary structure

SS confidence

Disorder: ? ?

Disorder confidence

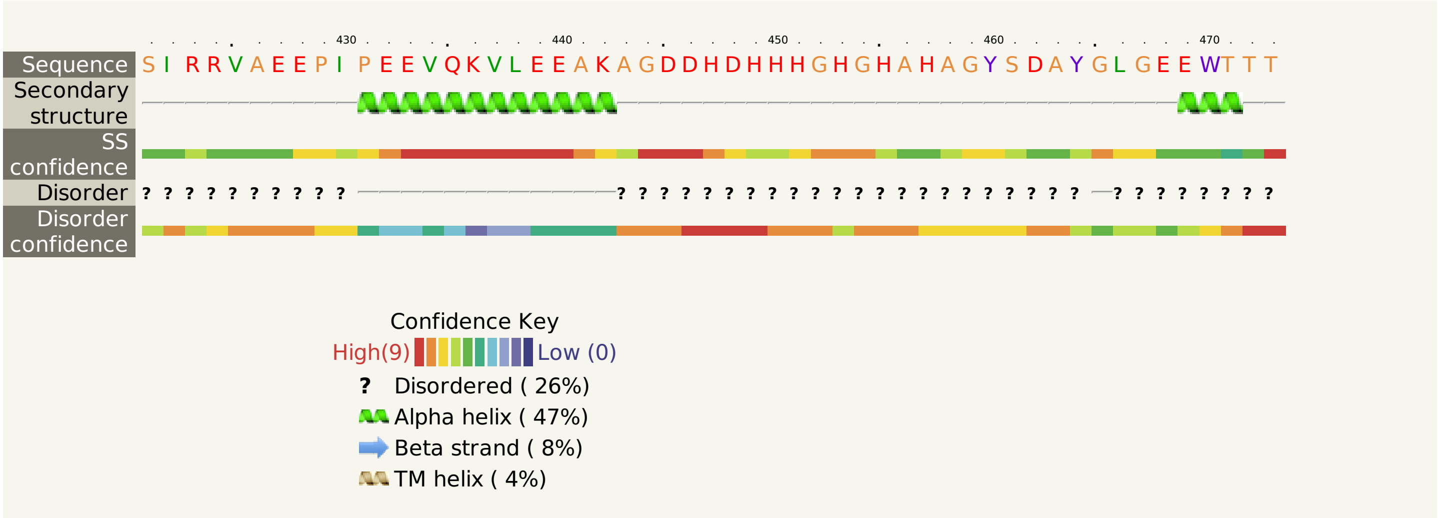
430
440
450
460
470
Sequence
S I R R V A E E P I P E E V Q K V L E E A K A G D D H D H H H G H G H A H A G Y S D A Y G L G E E W T T T
Secondary structure
SS confidence
Disorder
Disorder confidence
Confidence Key
High(9)
Low (0)
? Disordered (26%)
Alpha helix (47%)
Beta strand (8%)
TM helix (4%)
